# Graves’ disease in monozygotic twins – a case report

**DOI:** 10.1186/1472-6823-13-17

**Published:** 2013-05-25

**Authors:** Christoph A Rüst, Beat Knechtle, Thomas Rosemann

**Affiliations:** 1 Institute of General Practice and Health Services Research, University of Zurich, Zurich, Switzerland; 2 Gesundheitszentrum St. Gallen, Vadianstrasse 26, St. Gallen, 9001, Switzerland

## Abstract

**Background:**

Autoimmune thyroid diseases including Graves’ disease and Hashimoto’s thyroiditis are caused by immune response to self-thyroid antigens. The rare situation of hyperthyroidism with Graves’ disease in twins has been reported in a very few case reports in literature.

**Case presentation:**

We present monozygotic female twins developing consecutively Graves’ disease within five years. One year before the diagnosis of Graves’ disease was established in the first twin, the mother developed a toxic thyroid nodule with hyperthyroidism leading to hemi thyroidectomy. Both the mother and the twins were cigarette smokers. The twins were treated with carbamizole and this therapy led to normalization of thyroid stimulating hormone and thyroxine.

**Conclusion:**

This case report supports the hypothesis that a genetic factor as well as an environmental factor (cigarette smoking) might be of great importance in the aetiology of Graves’ disease.

## Background

The prevalence of thyroid disorders in the general population is small where hyperthyroidism seems to be less frequent than hypothyroidism. Kasagi et al. assessed thyroid function in the Japanese population [1]. Of the 1,818 examinees, 12 persons (0.7%) had manifest hypothyroidism, 105 persons (5.8%) showed subclinical hypothyroidism, 13 persons (0.7%) presented manifest thyrotoxicosis, and 39 persons (2.1%) suffered from subclinical thyrotoxicosis. Hollowell et al. investigated 16,533 people in the USA [2]. Hypothyroidism was found in 4.6% of the U.S. population (0.3% clinical and 4.3% subclinical) and hyperthyroidism in 1.3% (0.5% clinical and 0.7% subclinical).

Graves’ disease is a specific form of hyperthyroidism. Autoimmune thyroid diseases, including Graves’ disease and Hashimoto’s thyroiditis, are due to complex interactions between environmental and genetic factors. There are data from epidemiological, family, and twin studies demonstrating a strong genetic influence on the development of autoimmune thyroid diseases [3,4]. The rare situation of hyperthyroidism with Graves’ disease in twins has been reported in a very few case reports in literature [5-7]. Goldyn et al. described the serendipitous and simultaneous case of Graves’ disease in 6-year old female twins [5]. Mizukami et al. reported the case of Graves’ disease in identical twins with mental disorder [6]. Mimaru et al. described Graves’ disease in two pairs of female monozygotic twins [7]. They assumed that a genetic factor may be of great importance in the aetiology of Graves’ disease.

We present the case of monozygotic female twins developing consecutively Graves’ disease within five years. The mother of the twins was diagnosed one year before the diagnoses of Graves’ disease in the first twin with a toxic thyroid nodule with hyperthyroidism leading to hemi thyroidectomy.

## Case presentation

The description of this case report is in compliance with the Helsinki Declaration. In 2007, the mother of the unmarried twin A, who was born in 1980 and aged 26 at that time, noticed a swelling of her daughters’ neck. Later, she observed a set look. Due to these findings, she sent her daughter in April 2008 to a medical doctor who made the diagnoses of Graves’ disease based on the laboratory results (Table 1). A therapy with carbamizole was started with 30 mg per day and was then reduced to 10 mg per day. This therapy led to a normalization of the reduced TSH (thyroid stimulating hormone) and the increased

**Table 1:** Laboratory results upon diagnosis

	**Twin A**	**Twin B**
	**April 2008**	**January 2012**
TSH [0.25-4.0 mU/l]	< 0.003 *	< 0.004 *
fT_4_ [6.8-18 pmol/l]	72 *	190.3 *
fT_3_ [3.2-6.0 pmol/l]	7.8 *	6.5 *
TSH receptor antibodies [< 1.8 IU/l]	3.313 *	9.7 *
Thyroid Peroxidase Antibodies (TPOAb) [<60 U/ml]	570.9 *	4,944 *
Thyroglobulin Antibodies [<30 U/ml]	45.0 *	141.1 *

*TSH*: thyroid stimulating hormone, *fT*_*3*_: triiodothyronine, *fT*_*4*_: thyroxine. * = out of range.

**Figure 1:**
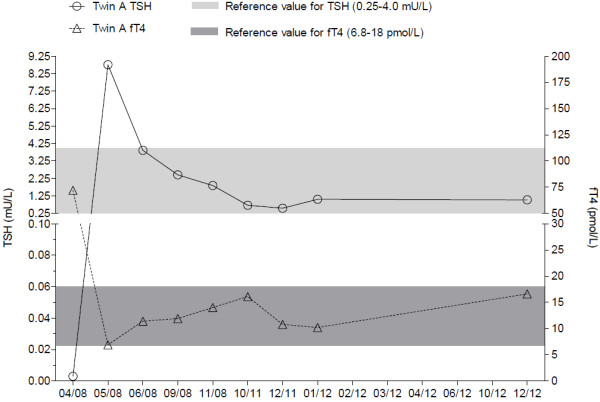
**Timeline of TSH and fT**_**4 **_**for twin A.**

 fT_4_ (thyroxine) (Figure 1). However, the compliance of carbamizole intake was weak and the dosage of carbamizole had to be changed several times. During drug therapy, the swelling of the neck became reduced. Due to further normalization of the laboratory results, the medication with carbamizole was stopped in autumn 2008. TSH and fT_4_ were measured later in irregular intervals due to mal-compliance of the patient. In the case history of twin A, tonsillectomy was performed in 1987, a pneumothorax in 2006 was treated conservatively, in 2007 she underwent hallux valgus correction and in 2008 varicose vein stripping. She started smoking cigarettes as a teenager and smokes 5–6 cigarettes a day.

**Figure 2:**
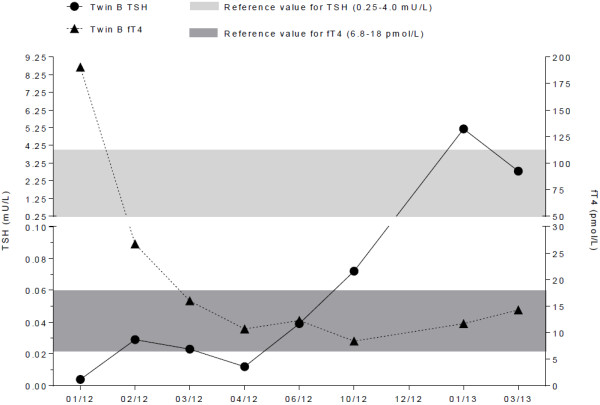
**Timeline of TSH and fT**_**4 **_**for twin B.**

In January 2012, the unmarried twin B aged 32 years went to the family doctor of twin A due to symptoms such as tiredness, dizziness, heart palpitations and sweating. The mother noticed a gaze. The patient herself remarked an increase in the collar size. Laboratory analyses revealed Graves’ disease (Table 1), and a treatment with carbamizole 20 mg per day was started. Due to normalization of fT_4_ (Figure 2) the dosage of carbamizole was reduced to 15 mg per day. The therapy was continued for one year. In the case history of twin B, varicose vein stripping was done in 2006. She also started smoking cigarettes as a teenager and smokes 5–10 cigarettes per day.

In the family history, the twin’s mother born in 1947 developed in 2006 at the age of 59 years a toxic thyroid nodule with hyperthyroidism leading to surgical therapy with hemi thyroidectomy. The resulting hypothyroidism was treated initially with 0.1 mg L-thyroxine and later with 0.05 mg L-thyroxine. In January 2012, she developed rheumatoid arthritis which was treated initially with prednisone and later with 20 mg leflunomide. Vitamin D deficiency was treated with 800 mg vitamin D daily. In the medical history of the mother, coronary

 artery disease and chronic obstructive pulmonary disease due to cigarette smoking with more than 50 pack years have to be noticed.

## Discussion and conclusions

Autoimmune thyroid diseases including Graves’ disease and Hashimoto’s thyroiditis are caused by immune response to self-thyroid antigens and affect approximately 2-5% of the general population [8,9]. Genetic susceptibility in combination with external factors such as smoking, viral/bacterial infection, and chemicals is believed to initiate the autoimmune response against thyroid antigens [3,8]. Graves’ disease is considered as a multifactorial disorder in which genetic susceptibility interacts with environmental and endogenous factors to cause disease [10].

Previous family and twin studies indicated that Graves’ disease has a heritable component [11-14]. Dittmar et al. reported that the risk for developing autoimmune thyroid diseases was 16-fold and 15-fold increased in children and siblings, respectively, of patients with autoimmune thyroid diseases [12]. The risk for developing Graves’ disease was enhanced 7-fold in both children and siblings. Family studies have also shown that some autoimmune disease cluster in families and genetic studies have been able to show shared susceptibility genes [11,13]. The highest familial risk for offspring of affected parents was noted for Graves’ disease with standardized incidence ratio (SIR) 3.87, followed by toxic nodular goitre (3.37) and nontoxic goitre (3.15). Familial risks were higher for affected siblings: toxic nodular goitre (11.66), Graves’ disease (5.51), and nontoxic goitre (5.40). Villanueva et al. studied 155 patients (131 with Graves’ disease and 24 with Hashimoto’s thyroiditis) with reliable information on the presence or absence of autoimmune thyroid disease in siblings [14]. Nine subjects had siblings with Graves’ disease and 13 subjects had siblings with Hashimoto’s thyroiditis.

Biometric twin modelling showed that approximately 75% of the total phenotypic variance in autoimmune thyroid disease was due to genetic effects [9,15,16]. The risk was higher for monozygotic than for dizygotic twins [17,18]. The aggregation of thyroid autoantibodies seemed to be genetically determined. Healthy first-degree relatives to patients with autoimmune thyroid disease showed a significant clustering of thyroid autoantibodies. Healthy monozygotic and dizygotic twin siblings to twins with autoimmune thyroid disease differed, however, in the prevalence of thyroid autoantibodies [18].

Apart from the present case and the recent case reports with Graves’ disease in twins [5-7], the combination of Graves’ disease and primary hypothyroidism in female twins has also been reported [19,20]. Aust et al. described twins where simultaneously Graves’ disease and Hashimoto’s thyroiditis occurred in monozygotic twins [19]. We report here the rare situation that hyperthyroidism was found in the mother of twins and both of her twin children. O’Connor et al. described a situation where premature twins of a mother with Graves’ disease developed concurrent hyperthyroidism and hypothyroidism [21]. In addition to twins with Graves’ disease, cases of subacute thyroiditis have been reported for siblings [22,23] and twins [24].

Both the twins and the mother were cigarette smoking. The impact of environmental triggers such as cigarette smoking, birth characteristics, infection with Yersinia enterocolitica, microchimerism and degree of X chromosome inactivation (XCI) has been evaluated by investigating autoimmune thyroid disease in twin pairs [16]. These studies indicate that smoking, Y. enterocolitica infection and skewed XCI may be causally associated with clinically overt autoimmune thyroid disease, but not with the presence of thyroid autoantibodies in euthyroid subjects [16].

A limitation of this case report is the fact that we do not distinguish between somatic mutations in toxic adenoma and germ cell mutations in autoimmune hyperthyroidism.

### Practical applications

Our case report gives some additional hints to a possible genetic susceptibility or at least genetic vulnerability for Grave’s disease in combination with external factors, such as smoking, viral/bacterial infection, and chemical agents.

### Consent

Written informed consent was obtained by both patients for the publication of this case report and any accompanying images.

## Competing interests

The authors have no conflict of interest in this work.

## Authors’ contributions

CAR drafted the manuscript, BK collected all medical reports of the patients and TR revised the manuscript critically for important intellectual content. All authors read and approved the final manuscript.

## Pre-publication history

The pre-publication history for this paper can be accessed here:

http://www.biomedcentral.com/1472-6823/13/17/prepub
